# Early-Onset Pectus Excavatum Is More Likely to Be Part of a Genetic Variation

**DOI:** 10.1055/a-2081-1288

**Published:** 2023-05-19

**Authors:** Ryan Billar, Stijn Heyman, Sarina Kant, René Wijnen, Frank Sleutels, Serwet Demirdas, J. Marco Schnater

**Affiliations:** 1 Department of Pediatric Surgery, Erasmus University Medical Center, Sophia Children's Hospital, Rotterdam, the Netherlands; 2 Department of Pediatric Surgery, ZNA, Antwerp Hospital Network, Queen Paola Children's Hospital, Antwerp, Belgium; 3 Department of Clinical Genetics, Erasmus University Medical Center, Rotterdam, the Netherlands

**Keywords:** surgery, chest anomaly, genetics, pediatrics

## Abstract

**Background** Potential underlying genetic variations of pectus excavatum (PE) are quite rare. Only one-fifth of PE cases are identified in the first decade of life and thus are of congenital origin. The objective of this study is to test if early-onset PE is more likely to be part of genetic variations than PE that becomes apparent during puberty or adolescence.

**Materials and Methods** Children younger than 11 years who presented with PE to the outpatient clinic of the Department of Pediatric Surgery at our center between 2014 and 2020 were screened by two clinical geneticists separately. Molecular analysis was performed based on the differential diagnosis. Data of all young PE patients who already had been referred for genetic counseling were analyzed retrospectively.

**Results** Pathogenic genetic variations were found in 8 of the 18 participants (44%): 3 syndromic disorders (Catel–Manzke syndrome and two Noonan syndromes), 3 chromosomal disorders (16p13.11 microduplication syndrome, 22q11.21 microduplication syndrome, and genetic gain at 1q44), 1 connective tissue disease (Loeys–Dietz syndrome), and 1 neuromuscular disorder (pathogenic variation in *BICD2* gene).

**Conclusion** Early-onset PE is more likely to be part of genetic variations than PE that becomes apparent during puberty or adolescence. Referral for genetic counseling should therefore be considered.

**Trial Registration:** NCT05443113

## Introduction

Pectus excavatum (PE) is a posterior depression of the sternum and costal cartilages, which produces a caved-in appearance of the anterior chest wall. 1 2 PE is the most common congenital chest wall deformity, and occurs approximately in 1 in every 300 to 1,000 live births with a male-to-female ratio of 4:1. 1 2

The possible underlying genetic mechanisms of PE are unknown. 3 4 5 A familial predisposition has been observed, and almost half of the patients have relatives with various skeletal variations. 6 7 However, a direct genetic link has not been found, and only a few pathogenic genetic variations have been identified to be associated with PE. 3 4 5 More importantly, several genetic disorders have been associated with PE; as these are relatively rare, it is thought unnecessary to refer every PE patient for genetic counseling. 6 8 9 10 The etiology of PE is poorly understood as well. The current leading theory describes an unbalanced overgrowth of the costochondral regions. 5 11 This unbalanced overgrowth especially occurs during the growth spurt and puberty, as a result of which the PE becomes more apparent or is first noted during this period. 5 12 Only 22% of all PE cases are identified in the first decade of life and thus are of congenital origin. 13 Therefore, younger PE patients are a unique patient group. 13 We hypothesize that early-onset PE is more likely to be part of a genetic variation than PE that becomes apparent not until during puberty or adolescence. PE being part of a genetic variation bears significance in regard to comorbidity and the reproductive choices related to the genetic variation. 10 14 Genetic variations also affect the surgical correction of PE, such as surgical technique, the timing of the correction, stabilization of the sternum, and long-term outcomes. 15 16 17 18 As part of standard care, we therefore have prospectively referred for genetic counseling of every young (< 11 years of age) PE patient who visited our outpatient clinic. In this study, we also retrospectively analyzed all young PE patients who had already been referred for genetic counseling.

## Materials and Methods

Between July 2014 and September 2020, all parents of pediatric PE patients aged younger than 11 years upon first visit to our outpatient clinic were contacted by telephone. Parents and patients were asked to visit the outpatient clinic for genetic counseling and undergo molecular analysis if deemed necessary. Written informed consent was obtained from the parents. Two clinical geneticists (S.D. and S.K.) performed the anamnesis (age, sex, family and medical history, intellectual and physical performance, and development) and physical examination via a standardized protocol (see Appendix A1 , available in the online version only) seperately. 4 Saliva was obtained from all patients and, if a genetic origin was suspected (based on the anamnesis, physical examination, and differential diagnosis), also from first-degree family members. Saliva was collected in Oragene DNA saliva collection kits (DNA Genotek, Inc., Ottawa, Ontario, Canada) and stored at room temperature (between 15 and 30°C). Both clinical geneticists, a pediatric thoracic surgeon (J.M.S.), and the researcher (R.B.) discussed all patients and their relatives. DNA analysis was performed (either single nucleotide polymorphism [SNP] array and/or exome sequencing filtered for a specific panel of genes) based on the differential diagnosis (see Appendix A2 , available in the online version only). Both clinical geneticists interpreted the genetic and molecular data. Anonymized data of young PE patients who had already been genetically analyzed before the start of this study were added to the analysis. Our main outcome was incidence of molecular variations in our cohort. Additionally, we evaluated the added clinical value of the standardized protocol “checklist referral of a patient with pectus excavatum for genetic counseling” by Billar et al. This protocol was created for the purpose of identifying those patients with PE who should be referred for genetic counseling (one or more major criteria or two or more minor criteria). 4 Applying this protocol, we identified all eligible patients in our study population. The added clinical value of using this protocol was determined by calculating the incidence of molecular variations in the identified patients compared with the number of missed diagnoses. This cohort study followed the STROBE guidelines (see Supplementary Material S1 ). This study has been approved by the Medical Review Ethics Committee of the Erasmus University Medical Center in Rotterdam prior to the beginning of the study (identification number MEC-2012–387) and has been registered at ClinicalTrials.gov (identifier: NCT05443113). This study was conducted according to the principles of the Declaration of Helsinki (64th WMA General Assembly, Fortaleza, Brazil, October 2013) and in accordance with the Dutch Medical Research Involving Human Subjects Act (Dutch: WMO).

**Table 1:** Patient characteristics arranged by ascending age on first presentation at the outpatient clinic

Patient no.	Sex	Age on first presentation of PE at outpatient clinic	Gestational age (wk + d)	Birth weight (g)	Pectus description	Age of pectus onset
**Patient 1**	Male	10 mo	41	3,820	Mild PE, low sternal	In the first few months
**Patient 2**	Female	11 mo	NR	NR	PE	NR
**Patient 3**	Male	1 y	NR	NR	Asymmetric PE	NR
**Patient 4**	Male	1 y, 11 mo	40 + 0	3,520	Low sternal, symmetrical deep	6 mo
**Patient 5**	Male	2 y, 4 mo	39 + 6	3,175	Low sternal, symmetrical deep	1 y
**Patient 6**	Female	3 y, 6 mo	40	4,030	PE, left-sided flaring	1 y
** Patient 7 a**	Male	3 y, 7 mo	40 + 5	4,000	Mild PE	2.5 y
**Patient 8**	Female	4 y, 4 mo	38	4,000	Deep PE	NR
**Patient 9**	Male	5 y	39	3,160	PE	NR
**Patient 10**	Male	5 y	NR	NR	Progressive PE	3 y
**Patient 11**	Male	5 y	35	2,200	Mild asymmetric PE, right deeper than left	In the first few months
**Patient 12**	Male	6 y, 9 mo	40 + 1	2,840	PE, broad thorax	NR
**Patient 13**	Female	7 y, 1 mo	37	3,400	Asymmetrical mild PE	NR
** Patient 14 a**	Female	8 y, 4 mo	39 + 2	3,300	PE, anterior positioned shoulders	Young age, exact age unknown
**Patient 15**	Female	9 y, 4 mo	40 + 1	3,500	PE with flaring of both sides	Young age, exact age unknown
**Patient 16**	Female	9 y, 10 mo	38 + 2	3,160	Mild PE, low sternal, no flaring	Young age, exact age unknown
**Patient 17**	Female	10 y, 4 mo	NR	NR	PE	NR
**Patient 18**	Male	10 y, 6 mo	NR	NR	Mild PE	NR

Abbreviations: NR, not reported; PE, pectus excavatum.

a Siblings.

**Table 2:** Results of molecular analysis

Patient no.	Prospective/retrospective	Differential diagnosis	Molecular analysis	Results
**Patient 1**	Prospective	No suspicion of underlying genetic disorder	Not performed	NA
**Patient 2**	Retrospective	Suspected of Noonan syndrome/RASopathy	KaryotypingSNP arraySanger sequencing and MLPA ( *SHOX* gene)MS-MLPA (Temple syndrome)WES (Noonan panel)	46, XX; normal karyotype, no sex chromosome anomaly or mosaicismarr[hg19](1–22,X)x2; normal profile with multiple ROH regionsNo *SHOX* sequence abnormalityNo abnormality in DNA methylation on 14q32Noonan syndrome, pathogenic variant in *SOS1* gene
**Patient 3**	Prospective	Suspect of an *FBN1* mutation, but informed consent was withdrawn for molecular analysis	Not performed	NA
**Patient 4**	Prospective	Suspect a connective tissue/collagenic disorder	WES (TAAD panel)	Loeys–Dietz syndrome, type 5, pathogenic variant in *TGFB3* gene
**Patient 5**	Prospective	Suspect of Noonan syndrome/RASopathy	MLPA for *NF1* , *SPRED1* , *BRAF* , *HRAS* , *KRAS* , and *NRAS* WES (Noonan panel)	No abnormalities in *NF1* , *SPRED1* , *BRAF* , *HRAS* , *KRAS* , *NRAS* No abnormalities, no reasons for additional molecular analysis
**Patient 6**	Prospective	Suspect of Noonan syndrome/RASopathy	MLPA for *NF1* , *SPRED1* , *BRAF* , *HRAS* , *KRAS* , and *NRAS* WES (Noonan panel)	No abnormalities in *NF1* , *SPRED1* , *BRAF* , *HRAS* , *KRAS* , *NRAS* No abnormalities, no reasons for additional molecular analysis
** Patient 7 a**	Prospective	Molecular analysis was postponed until molecular analysis of sister (patient 14) was performed. Later on, informed consent to perform molecular analysis was withdrawn	Not performed	NA
**Patient 8**	Retrospective	Suspect of Noonan syndrome/RASopathy	KaryotypingSNP arrayNGS panel (Noonan syndrome)	46, XX; normal karyotype, no sex chromosome anomaly or mosaicismarr[hg19](1–22,X)x2; normal profileNoonan syndrome, pathogenic variant in *PTPN11* gene
**Patient 9**	Retrospective	Very broad differential diagnosis due to multiple congenital anomalies	SNP arrayWhole-exome sequencing (MCA panel)	arr 16p13.11p12.3 × 3 pat; susceptibility locus associated with cognitive decline, autism, schizophrenia, heart and skeletal anomalies, craniosynostosis, and polydactylyLikely pathogenic variant in *BICD2* gene
**Patient 10**	Prospective	Suspect of microdeletion, Jeune syndrome, facioscapulohumeral muscular dystrophy	SNP arrayMetabolic screeningSanger sequencing ( *SMCHD1* gene)Repeat analysis of chromosome 4q35	arr[hg19](1–22)x2,(X,Y)x1; normal profileNo abnormalities in amino acid metabolism, mitochondrial fatty acid oxidation, no indication for an organic acidemia or congenital disorder of glycosylationNo *SMCHD1* sequence abnormalityNo abnormalities in repeats; no indication for *FSHD1*
**Patient 11**	Prospective	Suspect of Noonan syndrome/RASopathy	KaryotypingSNP arraySanger sequencing and MLPA ( *WT1* gene)MLPA for *NF1* , *SPRED1* , *BRAF* , *HRAS* , *KRAS* , *NRAS* WES (Noonan panel)	46,XY; normal karyotypearr 4q35.2 × 3,6q26 × 1; both the gain and loss are classified UV3No WT1 abnormalityNo abnormalities in *NF1* , *SPRED1* , *BRAF* , *HRAS* , *KRAS* , *NRAS* No abnormalities in Noonan panel
**Patient 12**	Retrospective	Microdeletion/duplication (22q11 deletion), Floating–Harbor syndrome	SNP array	arr[hg19] 1q44 × 3; gain at 1q44 classified as UV3
**Patient 13**	Retrospective	Very broad differential diagnosis due to multiple congenital anomalies	Metabolic screeningSNP array	No abnormalities in amino acid metabolism, mitochondrial fatty acid oxidation, no indication for an organic acidemia or congenital disorder of glycosylation16p13.11 microduplication syndrome
** Patient 14 a**	Prospective	Very broad differential diagnosis due to multiple congenital anomalies	SNP array	22q11.21 microduplication syndrome
**Patient 15**	Prospective	No suspicion of underlying genetic disorder	Not performed	NA
**Patient 16**	Prospective	Suspect of Waardenburg syndrome	WES (hearing impairment panel)	No abnormalities, no reasons for additional molecular analysis
**Patient 17**	Retrospective	Very broad differential diagnosis due to multiple congenital anomalies	SNP array,Sanger sequencing ( *FGFR3* gene)NGS panel (Ehlers–Danlos and Stickler syndromes)WES (MCA panel and full exome)	arr(1–22,X)x2; normal profileNo *FGFR3* sequence abnormalityNo abnormalities in gene panelCatel–Manzke syndrome, likely pathogenic homozygotic variant in the *TGDS* gene
**Patient 18**	Prospective	No suspicion of underlying genetic disorder	Not performed	NA

Abbreviations: MCA, multiple congenital anomaly; MLPA, multiplex ligation-dependent probe amplification; MS-MLPA, methylation-specific multiplex ligation-dependent probe amplification; NA, not applicable; NGS, next-generation sequencing; SNP array, single nucleotide polymorphism array; TAAD, thoracic aortic aneurysm and dissection and related disorders; WES, whole-exome sequencing.

a Siblings.

## Results

**Fig. 1:**
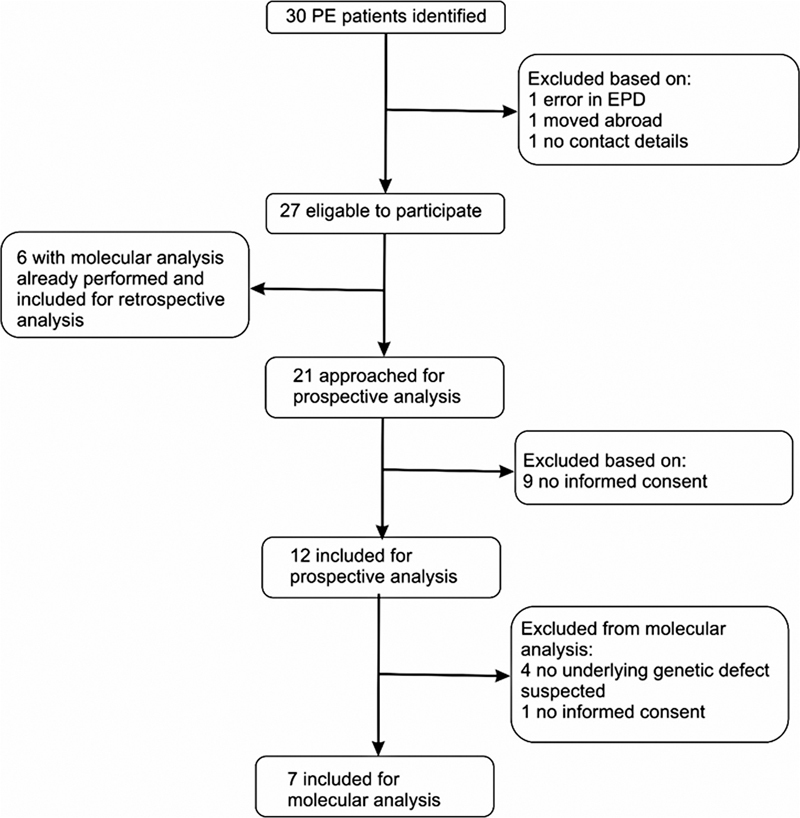
Flowchart of young (< 11 years) pectus excavatum (PE) patient identification and selection.

Thirty PE patients were identified meeting all inclusion criteria, of whom 12 were included in our prospective analysis and 6 were included in our retrospective analysis. Parents of 12 patients did not give informed consent and were therefore excluded from the analysis (see Fig. 1 ).

Detailed patient characteristics and PE characteristics are presented in Table 1 . Ten of the 18 participants (56%) were boys. In more than half of cases, the age of pectus onset was not exactly known, but must have been at a young age since the age on first presentation was less than 11 years, with a median age of 5 (interquartile range, 2.33–8.33) years. Birth parameters were mostly unremarkable; one patient had been born premature and one patient had been born dysmature.

Differential diagnoses were formulated based on the anamnesis, family history, and physical examination. Detailed data on the differential diagnosis of each patient and, if deemed necessary, the molecular analysis performed are presented in Table 2 . In four patients, no underlying genetic variation was suspected. In 1 out of the remaining 14 patients, an underlying genetic variation was suspected, but informed consent to perform molecular analysis was withdrawn. The 13 molecular analyses revealed 8 molecular variations: 3 syndromic disorders (1 case of Catel–Manzke syndrome 19 and 2 cases of Noonan syndrome), 3 chromosomal disorders (16p13.11 microduplication syndrome, 22q11.21 microduplication syndrome, and gain of chromosomal band 1q44), 1 connective tissue disease (Loeys–Dietz syndrome), and 1 neuromuscular disorder (pathogenic variation in *BICD2* gene). Of the eight patients in question, six had already been referred to a clinical geneticist. In one of these six patients, the clinical relevance of the found genetic variation was unknown (gain of 1q44), as the literature does not describe any clinical signs related to this variation. Informed consent to perform segregation analysis was withdrawn for this patient. Prematurity and dysmaturity were not predictive factors for genetic variations in our cohort.

The standardized protocol “checklist referral of a patient with pectus excavatum for genetic counseling” was applied to all 18 included patients ( Table 3 ). All six patients who had already been referred for genetic counseling were also identified by the standardized protocol. These six patients fulfilled at least one major criterion, of which “height less than 2 standard deviation score (SDS) or greater than 2 SDS” was the most common (i.e., in five of them). All of them had a molecular variation.

**Table 3:** Evaluation by standardized protocol “checklist referral of patient with pectus excavatum for genetic counseling”

	Criteria	Patient no.																	
		**1**	**2**	**3**	**4**	**5**	**6**	**7** a b	**8**	**9**	**10**	**11**	**12**	**13**	** 14 a**	**15**	**16**	**17**	**18**
Major	Positive first-degree family history for PE deformity and/or congenital cardiovascular anomalies				+	+	+	+							+	+			
	Height < 2 SDS or > 2 SDS		+					+	+	+			+					+	
	Intellectual disability/developmental delay or autism									+					+				
	Seizures																		
	Movement disorder																		
	Muscular hypotonia									+									
	Hearing loss																+		
	Craniosynostosis																		
	Low set ears								+	+									
	Down-slanting palpebral features									+	+								
	Cleft palate																	+	
	Short, webbed neck																		
	Hypoplasia of the pectoralis major																		
	Cardiovascular anomalies											+							
	Rib and spinal deformities																		
	Diaphragmatic hernia																		
	Kidney anomalies																		
	Limb joint contractures									+									
	Limb defects													+	+			+	
	Arachnodactyly or brachydactyly																		
	Anomalies of thumb and/or halluces				+										+				
	Loose redundant skin																		
	Three or more café-au-lait spots or lentigines																		
	History of pneumothorax																		
	Lung emphysema in childhood																		
	Malignancy																		
Minor	Positive second-degree family for pectus deformity and/or congenital cardiovascular anomalies					+													
	Dysmorphic facial features, score 1 for each (dysplastic ears or hypertelorism or malar hypoplasia or retrognathia and/or micrognathia or widow's peak or long facies or coarse facial features)				+				+						+				
	High arched palate										+								
	Increased span, limited elbow extension																		
	Joint hypermobility and/or dislocations				+													+	
	Pes planus						+				+				+				
	Shawl scrotum		NA				NA							NA	NA	NA	NA	NA	
	Cryptorchidism		NA				NA							NA	NA	NA	NA	NA	

Abbreviation: NA, not applicable; PE, pectus excavatum.

a Siblings.

b Minimal pectus and large idiopathic height.

Four prospectively included patients did not fit the criteria to be referred for genetic counseling. This was confirmed by the clinical geneticists, as in these patients no underlying genetic disorder was suspected and therefore molecular analysis was not deemed necessary. In one of these four patients, the PE was minimal and the patient's large height seemed to be idiopathic. The remaining eight prospectively included patients fulfilled at least one major criterion, and six of them fulfilled at least one minor criterion. The most common major criterion was “a positive first-degree family history for PE deformity and/or congenital cardiovascular anomalies” (6/8); the most common minor criterion was “dysmorphic facial features” (2/8). The incidence of molecular variations in these eight patients was 25% (2/8).

The overall incidence of molecular variations in all patients identified for referral for genetic counseling was 53% (8/15). No diagnoses were missed using this standardized protocol.

## Discussion

In this cohort of 18 young PE patients, 13 underwent molecular analysis, which revealed molecular variations in 8 cases (44%). This relatively high incidence suggests that young PE patients are more likely to be affected by underlying genetic variations. 20 21 The early recognition of these genetic variations in these eight patients has major implications regarding the comorbidity and reproductive choices, and the surgical correction of PE. 4 10 14 15 16 17 18 Be that as it may, in daily practice clinicians often miss an underlying genetic variation in PE patients, as representative clinical signs may be subtle or not even recognized. 8 22 Not to mention that approximately 40% of patients with chest wall deformities have family members with a chest wall deformity, implying an underlying genetic component. 7 13 23 Still, in most (familial) PE patients an underlying genetic variation is not found with molecular analysis, as potential underlying genetic variations are quite rare and a direct genetic link with PE has yet to be found. 5 7 23 This could be explained by multifactorial inheritance. 6

Furthermore, the rarity of potential underlying genetic variations makes it hard to select the appropriate molecular analysis. Performing an inappropriate type of analysis could result in misdiagnosis. 5 10 The rarity of underlying genetic variations is illustrated by Behr et al, who found that 5.3% of 187 PE patients in their cohort was affected by Marfan syndrome; the proportion is relatively high but still rare. 20 Similarly, in a study by Croitoru et al, 5.3% of 303 PE patients was diagnosed with Marfan syndrome and 2% with Ehlers–Danlos syndrome. 21 Furthermore, Kelly et al reported an incidence of 2.8% of Marfan syndrome in their cohort of 1,215 PE patients. 17 The above-described studies did not report the genetic analyses of diagnosed underlying genetic variations, nor were the diagnoses specified on age.

The rarity of these genetic variations makes it also redundant to refer every PE patient for genetic counseling. It is, therefore, desirable to set criteria for genetic counseling Our study suggests that onset of PE at a young age (< 11 years) is a decisive major criterion to refer a PE patient for genetic counseling. Prematurity and dysmaturity were not predictive factors for underlying genetic variations in our cohort.

Additionally, we have evaluated a standardized protocol that helps in identifying which patient with PE should be referred for genetic counseling. 4 Without this evaluation, identified underlying variations is in 8 of the 18 participants (44%). Still, on the basis of said evaluation, four patients could be excluded from genetic counseling, as an underlying genetic variation was not suspected. The yield therefore increased to 57% (8/14). In view of the high incidence of underlying genetic variations in young PE patients, we strongly advise to add to the “checklist referral of patient with pectus excavatum for genetic counseling” the major criterion “young age (< 11 years).”

## Limitations

The analyses of this cohort have several limitations. An underlying genetic variation was suspected in one PE patient, but informed consent to perform molecular analysis was withdrawn. This could have resulted in a potential underestimation of the number of underlying genetic variations in the prospectively analyzed patient group. Also, selection bias may be present in the retrospectively analyzed patient group, as some of these patients had first visited the clinical geneticist before they presented to the PE outpatient clinic. This could imply that these patients were more likely to be affected by an underlying genetic variation. Furthermore, the study population was limited, which could make the findings less generalizable. However, this limitation is debatable as young PE patients are in general rarely seen at outpatient clinics, which could potentially make our study cohort a representation of daily practice in other hospitals. Future research in collaboration with other PE expert centers is recommended to overcome such limitations.

## Conclusion

We found an incidence of pathogenic molecular variations of 44% in the study population of young-onset (< 11 years) PE patients, which suggests that these patients are more likely to be affected by an underlying genetic disorder. Furthermore, our data show that the application of the “checklist referral of patient with pectus excavatum for genetic counseling” could increase the yield of identifying underlying genetic disorders in young PE patients—and possibly in all PE patients. We strongly advise to add to this checklist “young age (< 11 years)” as a major criterion for referral.
