# Supplementary material for: Early-Onset Pectus Excavatum Is More Likely to Be Part of a Genetic Variation
Source: Eur J Pediatr Surg. 2023 May 19;34(4):325–32. doi: 10.1055/a-2081-1288 (PMC11226330; doi:10.1055/a-2081-1288)
Supplement: Supplementary file 1 — Supplementary Material [file 10-1055-a-2081-1288-s2023046557oa.pdf]

Appendix A1 Standardized assessment form

Areas	Major/minor, if applicable	Items	Comments
General		First presentation of PE at outpatient clinic	
		Patient no.	
		Sex	
		Age on first presentation of PE at outpatient clinic	
		Age on examination at clinical geneticist	
Pregnancy and delivery		Gestational age (wk + d)	
		Pregnancy course	
		Ultrasound findings	
		Birth weight (g)	
		Head circumference birth (cm + SDS)	
		Apgar score	
Developmental		Age rolling over in months	
		Age sitting in months	
		Age crawling in months	
		Age standing upright in months	
		Age walking independent in months	
		Age first word in months	
		Age combining 4–5 words in months	
		Type of school and grade	
		IQ (if available)	
Skeletal		Age pectus onset in years	
		Development pectus	
		Treatment pectus	
		Other skeletal deformations	
		Articular pain	
		Contractures	
		Other	
		Loss of strength	
		Sensibility	
		Spasticity	
		Stability while walking	
		Other	
Other organs		Intestine	
		Heart	
		Lungs	
		Liver	
		Spleen	
		Sight	
		Hearing	
		Anogenital	

(*Continued*)

 © 2023. The Author(s).

(*Continued*)

Areas	Major/minor, if applicable	Items	Comments
Parent–child resemblance		If yes: do parents have specific dysmorphisms	
Measurements		Height	
		Weight	
		Head circumference	
		Span	
		Span/height ratio	
		Sitting height	
		Right hand palm + fingers	
		Dig 3 right hand	
		ICD and OCD	
Face/neck		Head shape	
		Hairline	
		Neck	
		Ears	
		Eyes	
		Nose	
		Lips	
		Chin	
		Other	
Thorax		Pectus description	
		Nipples	
		Other	
Abdomen		Hepato-/splenomegaly	
		Other	
Extremities		Hands	
		Arms	
		Feet	
		Legs	
		Beighton score	
		Wrist sign	
		Thumb sign	
		Other	
Anogenital		Anogenital abnormalities	
Neurological		Tonus	
		Strength	
		Sensibility	
		Cranial nerves	
		Hypoplasia muscles	
Skin		Lentigines	
		Café-au-lait spots	
		Other discolorations	
		Skin laxity (measured at the ventral side of the wrist, max 1.5 cm)	
		Other	

 © 2023. The Author(s).

(*Continued*)

Areas	Major/minor, if applicable	Items	Comments
Checklist referral of a patient with pectus excavatum for genetic counseling	Major criteria	Positive first degree family history for PE deformity and/or congenital cardiovascular anomalies	
		Height < 2 SDS or > 2 SDS	
		Intellectual disability/developmental delay or autism	
		Seizures	
		Movement disorder	
		Muscular hypotonia	
		Hearing loss	
		Craniosynostosis	
		Low set ears	
		Down-slanting palpebral features	
		Cleft palate	
		Short webbed neck	
		Hypoplasia of the pectoralis major	
		Cardiovascular anomalies	
		Rib and spinal deformities	
		Diaphragmatic hernia	
		Kidney anomalies	
		Limb joint contractures	
		Limb defects	
		Arachnodactyly or brachydactyly	
		Anomalies of thumb and/or halluces	
		Loose redundant skin	
		Three or more café-au-lait spots or lentigines	
		History of pneumothorax	
		Lung emphysema in childhood	
		Malignancy	
	Minor criteria	Positive second degree family for pectus deformity and/or congenital cardiovascular anomalies	
		Dysmorphic facial features, score 1 for each (dysplastic ears or hypertelorism or malar hypoplasia or retrognathia and/or micrognathia or widow's peak or long facies or coarse facial features	
		High arched palate	
		Increased span, limited elbow extension	
		Joint hypermobility and/or dislocations	
		Pes planus	
		Shawl scrotum	
		Cryptorchidism	

 © 2023. The Author(s).

Appendix A2 Additional Materials and Methods

Karyotyping

GTG banded karyotyping performed on metaphase spreads harvested from lymphocytes.

SNP array:

Global screening array (or comparable) performed with BeadArray technology (Illumina).

ROH; region of homozygosity

WES:

Whole-exome sequencing (WES) applied on SureSelect Clinical Research Exome (or comparable) captured CDS regions (Agilent) and sequenced using NGS platforms (Illumina). In downstream analysis, data are filtered using certain gene panels (e.g., Noonan, multiple congenital anomaly [MCA], hearing impairment). For details on the gene content of the panels: https://www.erasmusmc.nl/nl-nl/patientenzorg/laboratoriumspecialismen/klinische-genetica#35d085e6-2dc0-48a3-9dfd-8aa502ca959e

MLPA and MS-MLPA:

Multiplex ligation-dependent probe amplification (MLPA) to detect specific small chromosomal abnormalities (i.e., single or partial exon/gene deletions). Methylation-specific MLPA to detect DNA methylation abnormalities. Performed according to manufacturer (MRC Holland).

NGS-panels:

Noonan: Custom capture of CDS for genes: *d A2ML1*, *BRAF*, *CBL*, *HRAS*, *KRAS*, *MAP2K1*, *MAP2K2*, *NRAS*, *RAF1*, *RIT1*, *SOS1*, *SHOC2*, and *SPRED1*.

Ehlers-Danlos and Stickler syndromes: Custom capture of CDS for genes: *ADAMTS2*, *B3GALT6*, *B4GALT7*, *B3GAT3*, *CHST3*, *CHST14*, *COL1A1*, *COL1A2*, *COL12A1*, *COL5A1*, *COL5A2*, *DSE*, *FKBP14*, *FLNB*, *PLOD1*, *PLOD3*, *PRDM5*, *SLC39A13*, *TNXB*, *ZNF469*, *COL3Al*, *COL2A1*, *COL11A1*, *COL11A2*, *COL9A1*, *COL9A2*, *COL9A3*, *SLC26A2*, *VCAN*.

 © 2023. The Author(s).

Supplementary Material S1 STROBE statement—checklist of items that should be included in reports of cohort studies

	Item No.	Recommendation	Page No.
**Title and abstract**	1	(a) Indicate the study's design with a commonly used term in the title or the abstract	1, 5
		(b) Provide in the abstract an informative and balanced summary of what was done and what was found	5
**Introduction**			
Background/rationale	2	Explain the scientific background and rationale for the investigation being reported	6, 7
Objectives	3	State specific objectives, including any prespecified hypotheses	6, 7
**Methods**			
Study design	4	Present key elements of study design early in the paper	8
Setting	5	Describe the setting, locations, and relevant dates, including periods of recruitment, exposure, follow-up, and data collection	8
Participants	6	(a) Give the eligibility criteria, and the sources and methods of selection of participants. Describe methods of follow-up	8
		(b) For matched studies, give matching criteria and number of exposed and unexposed	NA
Variables	7	Clearly define all outcomes, exposures, predictors, potential confounders, and effect modifiers. Give diagnostic criteria, if applicable	8, 9
Data sources/ measurement	8	For each variable of interest, give sources of data and details of methods of assessment (measurement). Describe comparability of assessment methods if there is more than one group	8, 9
Bias	9	Describe any efforts to address potential sources of bias	NA, stated in discussion
Study size	10	Explain how the study size was arrived at	8
Quantitative variables	11	Explain how quantitative variables were handled in the analyses. If applicable, describe which groupings were chosen and why	8, 9
Statistical methods	12	(a) Describe all statistical methods, including those used to control for confounding	NA
		(b) Describe any methods used to examine subgroups and interactions	
		(c) Explain how missing data were addressed	
		(d) If applicable, explain how loss to follow-up was addressed	
		(e) Describe any sensitivity analyses	
**Results**			
Participants	13*	(a) Report numbers of individuals at each stage of study, e.g., numbers potentially eligible, examined for eligibility, confirmed eligible, included in the study, completing follow-up, and analyzed	10
		(b) Give reasons for non-participation at each stage	10 and CONSORT flow chart
		(c) Consider use of a flow diagram	See Fig. 1, CONSORT flow chart

(*Continued*)

 © 2023. The Author(s).

(*Continued*)

	Item No.	Recommendation	Page No.
Descriptive data	14*	(a) Give characteristics of study participants (e.g., demographic, clinical, social) and information on exposures and potential confounders	8 and Table 1
		(b) Indicate number of participants with missing data for each variable of interest	Table 1
		(c) Summarize follow-up time (e.g., average and total amount)	NA
Outcome data	15*	Report numbers of outcome events or summary measures over time	10, 11
Main results	16	(a) Give unadjusted estimates and, if applicable, confounder-adjusted estimates and their precision (e.g., 95% confidence interval). Make clear which confounders were adjusted for and why they were included	NA
		(b) Report category boundaries when continuous variables were categorized	
		(c) If relevant, consider translating estimates of relative risk into absolute risk for a meaningful period	NA<br>NA
Other analyses	17	Report other analyses done, e.g., analyses of subgroups and interactions, and sensitivity analyses	NA
**Discussion**			
Key results	18	Summarize key results with reference to study objectives	12, 13
Limitations	19	Discuss limitations of the study, taking into account sources of potential bias or imprecision. Discuss both direction and magnitude of any potential bias	13, 14
Interpretation	20	Give a cautious overall interpretation of results considering objectives, limitations, multiplicity of analyses, results from similar studies, and other relevant evidence	12, 13, 14
Generalizability	21	Discuss the generalizability (external validity) of the study results	13, 14
**Other information**			
Funding	22	Give the source of funding and the role of the funders for the present study and, if applicable, for the original study on which the present article is based	2

 © 2023. The Author(s).
